# Liquid Chromatography-Mass Spectrometry (LC-MS) Derivatization-Based Methods for the Determination of Fatty Acids in Biological Samples

**DOI:** 10.3390/molecules27175717

**Published:** 2022-09-05

**Authors:** Christiana Mantzourani, Maroula G. Kokotou

**Affiliations:** Laboratory of Chemistry, Department of Food Science and Human Nutrition, Agricultural University of Athens, Iera Odos 75, 11855 Athens, Greece

**Keywords:** charge reversal, derivatization reagents, fatty acids, liquid chromatography, mass spectrometry

## Abstract

Fatty acids (FAs) play pleiotropic roles in living organisms, acting as signaling molecules and gene regulators. They are present in plants and foods and may affect human health by food ingestion. As a consequence, analytical methods for their determination in biological fluids, plants and foods have attracted high interest. Undoubtedly, mass spectrometry (MS) has become an indispensable technique for the analysis of FAs. Due to the inherent poor ionization efficiency of FAs, their chemical derivatization prior to analysis is often employed. Usually, the derivatization of the FA carboxyl group aims to charge reversal, allowing detection and quantification in positive ion mode, thus, resulting in an increase in sensitivity in determination. Another approach is the derivatization of the double bond of unsaturated FAs, which aims to identify the double bond location. The present review summarizes the various classes of reagents developed for FA derivatization and discusses their applications in the liquid chromatography-MS (LC-MS) analysis of FAs in various matrices, including plasma and feces. In addition, applications for the determination of eicosanoids and fatty acid esters of hydroxy fatty acids (FAHFAs) are discussed.

## 1. Introduction

Fatty acids (FAs) constitute one of the most important classes of lipids, and they are ubiquitous in every living organism. They are natural carboxylic acids with aliphatic chains, mostly found in esterified form as triglycerides (triacylglycerols, TAGs) or phospholipids, but also in their free form as carboxylic acids. In humans, they are present in biological fluids (for example, human plasma) and in various tissues. However, they are also present in almost every natural source, including plants and foods. They can be classified in various classes, depending on their chemical structural characteristics. According to their chain length, they are classified into short-chain FAs (SCFAs, 2–6 carbon atoms); medium-chain FAs (MCFAs, 7–12 carbon atoms); long-chain FAs (LCFAs, 13–22 carbon atoms); and very-long-chain FAs (VLFAs, >22 carbon atoms). According to their saturation degree, they are classified into saturated FAs (SFAs); monounsaturated FAs (MUFAs, a single double bond, usually *cis*); and polyunsaturated FAs (PUFAs, several double bonds, usually *cis*). PUFAs are further divided into ω-6 (or n-6) and ω-3 (or n-3) FAs, based on the distance of the first double bond from the terminal carbon atom.

In addition to their roles as structural elements in cell membranes as phospholipids or as the energy reservoir in adipose tissue in the form of TAGs, FAs play diverse roles in human health, acting as signaling molecules and gene regulators [1,2]. Intracellular free FAs (FFAs) together with their metabolites are able to affect gene expression upon interaction with the nuclear peroxisome proliferator-activated receptors (PPARs) [3], while several seven-transmembrane domain receptors have also been identified for the direct interaction of various classes of FFAs with them [4]. Long-chain FFAs are ligands for the FFA1 receptor (also known as GPR40) and FFA4 receptor (also known as GPR120), which indirectly affect energy homeostasis via hormonal signaling, thus, linking FAs and diet with metabolic disorders and type 2 diabetes (T2D) [5,6]. Short-chain FFAs bind to the FFA2 receptor (also known as GPR41) and FFA3 receptor (also known as GPR43), which are related to the gut-microbiome-mediated effects on health [7].

Apart from the common FAs, a great number of FAs bearing additional functional groups are present in many natural sources and living organisms. Such uncommon (rare) FAs are usually found at low concentrations; however they exert interesting bioactivities. These uncommon FAs include odd-chain, branched and cyclic FAs [8] and FAs bearing functional groups such as hydroxy [9,10,11,12], oxo [13,14], epoxy [15,16] and nitro [17,18].

**Figure 1:**
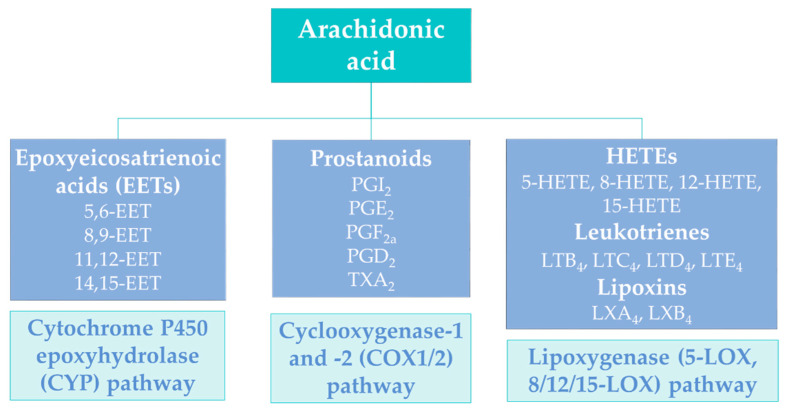
Generation of eicosanoid mediators from arachidonic acid.

Another very important class of lipidic carboxylic acids, which has attracted high medicinal interest, is the class of eicosanoids. These bioactive metabolites are generated by the oxidation of arachidonic acid, following its release from membrane glycerophospholipids by the enzymatic action of phospholipase A_2_ (PLA_2_) [19]. As shown in Figure 1, a variety of lipid messengers are produced by the activity of various enzymes on arachidonic acid, leading to compounds known as prostaglandins, leukotrienes, etc. [20]. All these carboxylic acids are involved in inflammatory diseases, including atherosclerosis, diabetes and cancer [21].

**Figure 2:**
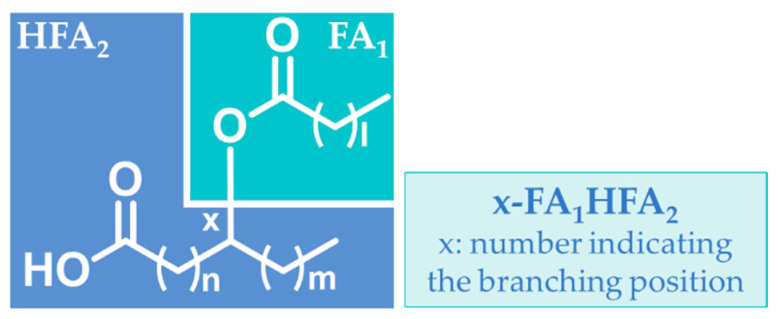
Structure of fatty acid esters of hydroxy fatty acids (FAHFAs).

Recently, a new class of endogenous bioactive lipids has been identified, which is known as fatty acid esters of hydroxy fatty acids (FAHFAs) [22]. Each of the various classes of FAHFAs consist of multiple regio-isomers. As shown in the general structure of FAHFAs depicted in Figure 2, the hydroxy group connecting the hydroxy FA with the other FA chain by an ester bond may be at different positions (e.g., 5- or 9-). The bioactivities of FAHFAs and the methods for their analysis are summarized in recent review articles [23,24].

Due to the great importance of FAs and related lipid compounds such as eicosanoids and FAHFAs as bioactive agents and food ingredients, their determination in various matrices, including biological fluids, plants and foods, is of high interest. The classical method for the determination of FAs in various samples employs the use of gas chromatography combined with either flame ionization detection (GC-FID) or mass spectrometry detection (GC-MS), which requires the conversion of FAs into the corresponding methyl esters (FAMEs) [25,26]. However, the conversion of FAs to FAMEs has also been used for the analysis of monounsaturated branched chain FAs by electron ionization and covalent adduct chemical ionization tandem MS [27]. Dennis and coworkers presented a highly sensitive quantitative lipidomics analysis of FAs in biological samples by GC-MS, involving the conversion of FAs into corresponding esters by treatment with pentafluorobenzyl bromide [28]. Adopting various analytical methods, they demonstrated that lipidomics reveals a remarkable diversity of lipids in human plasma [29].

Liquid chromatography-mass spectrometry (LC/MS) methods for the determination of FAs in various samples have also been developed, involving or avoiding a derivatization step. Koletzko et al. developed a straightforward quantitative LC–tandem mass spectrometry (LC–MS/MS) method for the determination of non-esterified FAs (NEFAs) in plasma [30]. Masoodi et al. described a high-throughput quantitative lipidomics analysis of NEFAs in plasma by LC coupled to high-resolution mass spectrometry (LC-HRMS) [31]. Positional isomers such as polyunsaturated and branched-chain species were sufficiently separated and the possibility to perform untargeted screening was also demonstrated. Recently, we presented LC-HRMS methods for the rapid and direct determination of FFAs in milk [32] and human plasma from healthy and diabetic subjects [33]. Thus, the direct determination of common FFAs is possible by avoiding derivatization. However, when FFAs are present in samples at very low concentrations, for example in the case of eicosanoids or FAHFAs, methods offering high sensitivity are required.

**Figure 3:**
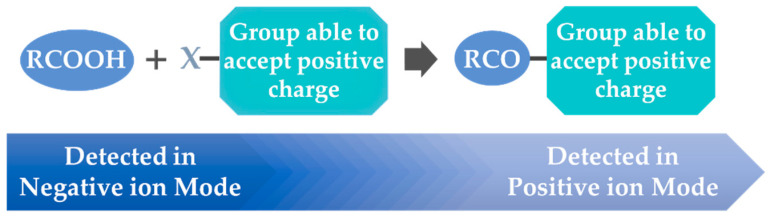
Basic principle of FA derivatization for its charge reversal.

Although FAs can be analyzed as [M–H]^−^ in negative ion mode using electrospray ionization (ESI) or atmospheric pressure chemical ionization (APCI), as presented above in the previous representative examples, in some cases, insufficient ionization in negative ion mode has been observed, possibly due to the moderate gas-phase acidity of the carboxy group. This may result in limited analytical sensitivity. In addition, the characteristic and sufficient fragmentation of both saturated and unsaturated FAs is not usually observed via collision-induced dissociation [34]. To overcome these drawbacks, a strategy has been developed, which employs the charge reversal of the analytes by derivatization of the carboxyl group and conversion to a derivative, which can easily accept positive charge (Figure 3). Thus, MS may operate in positive ion mode, monitoring [M+H]^+^ [34,35].

Another derivatization approach is the functionalization of the double bond of unsaturated FAs, which is discussed in Section 3. Such a derivatization allows the identification of the position of the double bond in a lipidic chain.

The aim of the present review article is to summarize the reagents which have been used for the derivatization of FAs and, in particular, those used in order to reverse their charge and make them suitable for MS detection and quantification in positive ESI mode, instead of negative ESI mode. The derivatization reagents are categorized on the basis of their chemical structure and their functional groups. Various applications of these derivatization approaches for the determination of FAs in biological samples are discussed. The most recent advances in this growing field are summarized in the present review article, including the very recently reported approaches for the determination of the double bond location in FAs.

## 2. Derivatization of Carboxyl Group—Classes of Derivatizing Agents

### 2.1. Primary Amines

**Figure 4:**
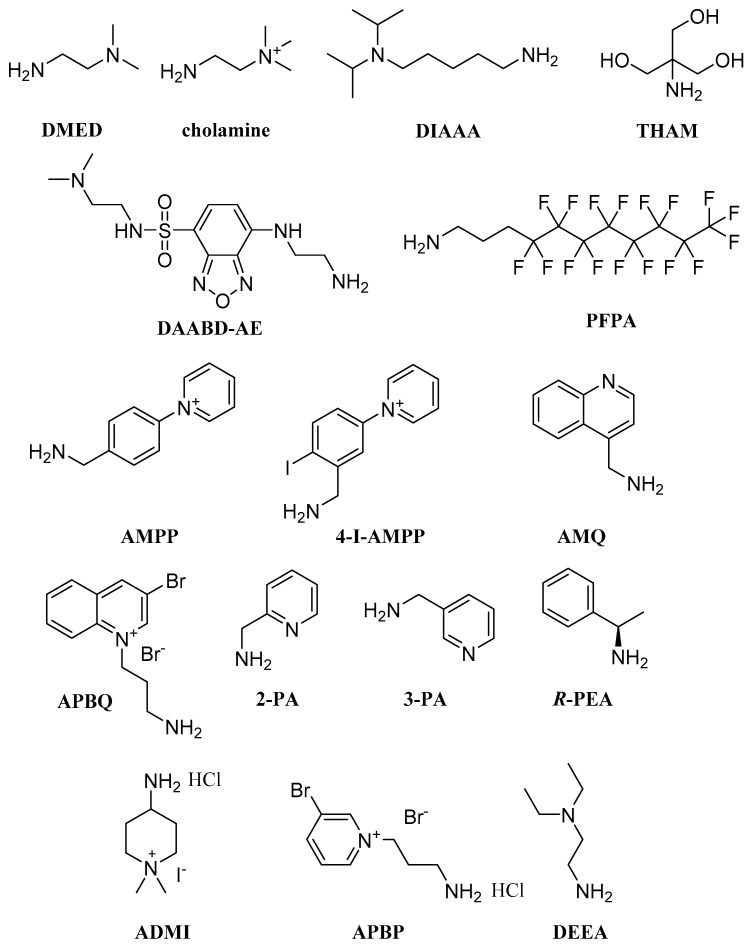
Primary amines as derivatization reagents for LC-HRMS analysis of FFAs.

Coupling with primary amines has been a very popular approach in FA derivatization reactions for LC-MS analysis. This is attributed to the nucleophilicity of primary amines, which can result in the quantitative formation of amides under mild conditions. Numerous reagents have been developed for this purpose, bearing additional functional groups for charge reversal, mainly tertiary amines. Typical examples are primary aliphatic amines with simple structures such as DMED (2-dimethylaminoethylamine) (Figure 4) [36,37]. Zhu et al. used DMED and *d_4_*-DMED (*d_4_*-2-dimethylaminoethylamine) in eicosanoid labeling. The detection sensitivities of DMED labeled eicosanoids showed a 5–138-fold improvement in serum matrix compared to unlabeled analytes, and a good separation of different isomers was accomplished [36]. Later, the same team developed another method for the quantification of FAHFAs (PAHSAs, OAHSAs, SAHSAs and POHSAs) in various biological samples based on a SAX-SPE-CL-UHPLC–MS/MS analysis. Due to anion exchange interactions between the SAX cartridge and the analytes, the FAHFAs were selectively extracted and purified, while the DMED labeling enhanced their detection sensitivities in a UHPLC–MS analysis. The corresponding deuterium-labeled derivatives were used as internal standards and this method was implemented on the quantification of seven endogenous FAHFAs in different organs and tissue of rats and human serum [37]. Additionally, a DMED-FAHFA in silico library of 4290 high-resolution tandem mass spectra from 264 different FAHFA classes was constructed based on the MS/MS fragmentation patterns of DMED-FAHFA authentic standards, which were applied to computer-generated DMED-FAHFAs [38]. Recently, the same team established a derivatization-based in-source fragmentation-information-dependent acquisition (DISF-IDA) strategy for the profiling of submetabolomes by LC-ESI-Q-TOF MS. In particular, 36 carboxylated compounds labeled with DMED were used as model compounds for the profiling of carboxylated submetabolome in mice feces [39]. In 2020, DMED was also utilized for the labeling of short chain fatty acid esters of hydroxy fatty acids (SFAHFAs). With their method, Gowda et al. were able to identify new SFAHFAs in rat colon contents with high mass accuracy using UHPLC/LTQ Orbitrap MS in positive ion ESI mode with collision-induced dissociation (CID) [40]. In 2022, Gowda et al. also presented a sensitive method for the quantification of SCFAs and their hydroxyl derivatives, by LC–MS/MS, once again employing DMED as a derivatization reagent. The method was validated by analyzing spiked intestinal content samples, with limits of detection and quantification of SCFAs at 0.5 and 5 fmol, respectively [41].

Bian et al. developed a method for determining LCFAs in biological samples, including a derivatization step with cholamine (Figure 4), using HATU as the coupling reagent in the presence of HOBt. A 2000-fold increase in sensitivity was observed in comparison to the non-derivatization method, as well as an enhanced ionization and LCFA separation [42]. In 2018, the same group compared different primary amines for the derivatization of multiple carboxyl-containing metabolites. As a result, DIAAA (5-(diisopropylamino)amylamine) (Figure 4) proved to be the best choice, allowing the simultaneous determination of SCFAs and LCFAs among other metabolites, with satisfactory separation resolution using UHPLC-Q-TOF/MS [43]. Recently, Yang et al. developed a method for the quantitative analysis of n-3 PUFAs using cholamine-*d_0_* and cholamine-*d_9_* as labeling agents for biological samples and internal standards, respectively. Their method was applied to mouse serum and brain tissue with improved MS sensitivity and chromatographic separation [44].

Another approach in FA derivatization is the conversion to bis(hydroxymethyl) oxazoline derivatives using THAM (tris(hydroxymethyl)aminomethane) (Figure 4). Williams et al. established a solvent free, microwave assisted reaction for the conversion of FAs to the corresponding 2-oxazoline products in a single step. Using LC-APCI-MS, a 200-fold improvement in the limit of quantitation (LOQ) for palmitic and oleic acid was observed, and a 2-fold improvement for arachidonic acid [45]. Additionally, primary amines containing benzofurazan moieties were synthesized and utilized for a LC-MS/MS analysis of FAs. Specifically, DAABD-AE ({4-[2-(*N*,*N*-dimethylamino)ethylaminosulfonyl]-7-(2-aminoethylamino)-2,1,3-benzoxadiazole}) (Figure 4) was applied to an FA analysis in rat plasma samples. The derivatization reaction took place at 60 °C for 30 min and the derivatized FAs were separated on a reversed-phase column, with LODs in the femtomole range [46]. In 2020, Zheng et al. developed a fluorous derivatization method for the quantification of LCUFAs in biological samples. LCUFAs were perfluoroalkylated with PFPA (3-(perfluorooctyl)-propylamine) (Figure 4) and retained on a fluorous phase LC column, achieving a limit of detection (LOD) at an atto-molar level and determining eight LCUFAs with high sensitivity [47].

Other primary amines that have been employed for the derivatization of FAs bear pyridine or quinoline moieties that can be easily protonated. For example, AMPP (*N*-(4-aminomethylphenyl)-pyridinium) (Figure 4) is a common derivatization reagent containing a pyridine moiety. Bollinger et al. first described the synthesis of AMPP in 2010, reporting that the derivatization of eicosanoids with AMPP improved the sensitivity of detection by 10- to 20-fold compared to the negative mode ESI detection of the underivatized analytes. This protocol was then used for the detection of eicosanoids in complex biological samples with LOQs in the 200–900 fg range [48]. The same team later demonstrated that coupling FAs with AMPP using EDCI as a coupling reagent resulted in a 60000-fold increase in sensitivity in comparison to the non-derivatization method. Moreover, analytical specificity was improved due to the significant fragmentation of the precursor ions, and their method was successfully applied to mouse serum [49]. Liu et al. used AMPP in their charge-switch derivatization protocol in an effort to label linoleic acid, arachidonic acid and docosahexaenoic acid metabolites. Their method yielded a 10- to 30-fold increase in ionization efficiency, with LOQs ranging between 0.05 and 6.0 pg [50]. Furthermore, Narreddula et al. developed dual-function derivatization reagents based on AMPP, in order to improve FA detection. The 4-I-AMPP (1-(3-(aminomethyl)-4-iodophenyl)pyridin-1-ium) (Figure 4) derivatives include a selectively photoactivated aryl-iodide moiety, in order to generate structurally diagnostic ions via radical-directed dissociation. The derivatization of diverse FA structures yielded photodissociation mass spectra with characteristic radical-driven fragmentation patterns, enabling the distinction of isomers [51]. On the other hand, one derivatization reagent that contains a quinoline moiety is AMQ (4-aminomethylquinoline) (Figure 4). AMQ has been used in SCFA analysis in human fecal samples by UPLC/MS/MS. Fu et al. published a new method with a short reaction and analysis time, which enabled the quantitation of SCFAs with improved sensitivity [52]. Finally, APBQ (1-(3-aminopropyl)-3-bromoquinolinium bromide) (Figure 4) is a reagent that possesses a permanent positive charge as well as a bromine atom in its structure. Mochizuki et al. achieved a qualitative and quantitative determination of a series of FAs in human plasma and saliva after derivatization with APBQ, taking advantage of the bromine isotope pattern and the improved sensitivity caused by the permanent positive charge [53].

Picolylamines are primary amines attached to a pyridine ring, a characteristic that renders them promising agents for FA tagging. 3-PA (3-picolylamine) (Figure 4) was employed by Li et al. in a derivatization method for FA analysis using orbitrap mass spectrometry in positive ESI mode, which provided enhanced sensitivity and selectivity. They reported that this method had an LOD in the low femtomole range, and 14 saturated and unsaturated FAs were separated in a 15 min run [54]. In addition, Nagatomo et al. used 2-PA (Figure 4) in their method for the determination of 10 SCFAs in the fecal samples of obese type II diabetes mice [55]. Lastly, Wu et al. also used 2-PA for the derivatization of FAs and other metabolites in seminal plasma samples. With their developed method, they achieved a 44 to 1500-fold enhancement of FA signals in positive ESI mode [56].

Interestingly, (*R*)-(+)-1-phenylethylamine (*R*-PEA) (Figure 4) has been also tested as a derivatization reagent for FFA detection in soil samples. Soil sample extracts were first treated with ethyl chloroformate and triethylamine to afford anhydride intermediates that were then reacted with *R*-PEA. This protocol resulted in chiral FA derivatives that could be separated with the use of chiral chromatographic columns [57]. In 2022, Zhu et al. developed a two-step derivatization strategy for the identification of 2/3-OHUFAs and 2/3-OHFAs. The first step was the derivatization with ADMI (4-amino-1,1-dimethylpiperidin-1-ium iodide hydrochloride) (Figure 4), followed by mCPBA-oxidation. This protocol enabled the fast and accurate determination of 2/3-OHUFAs and 2/3OHFAs in complex matrices without the use of standards and was applied in a mouse melanoma model analysis [58]. Another interesting approach involves the use of derivatization reagents that contain a bromine atom. In particular, APBP (1-(3-aminopropyl)-3-bromopyridinium bromide hydrochloride) (Figure 4) has been used as a labeling agent, and a subsequent cluster analysis of the derivatized mixture of compounds can be employed in order to resolve the individual derivatized species [59]. Recently, an isotope-free method with dual derivatization using LC-MS was reported for the quantification of SCFAs. According to this method, DMED or Dns-HZ (*N*,*N*-dimethyldansulfonyl hydrazide) were used to label the samples, and their structural analogues DEEA (*N*,*N*-diethyl ethylene diamine) (Figure 4) or Dens-HZ (*N*,*N*-diethyldansulfonyl hydrazide) tagged standard mixtures. Specifically, DMED/DEEA was employed for the dual derivatization and quantification of fecal SCFAs from hepatocellular carcinoma patients and healthy individuals [60].

### 2.2. Secondary Amines

**Figure 5:**
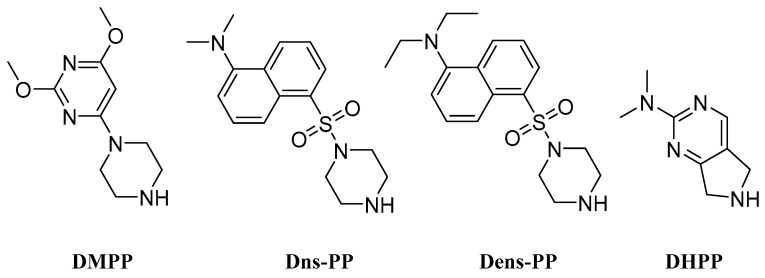
Secondary amines as derivatization reagents for LC-HRMS analysis of FFAs.

To increase the hydrophobicity and retention times of analytes in biological samples, various secondary amines have been designed and tested as derivatization reagents for FA analysis. As an example, DMPP (2,4-dimethoxy-6-piperazin-1-yl pyrimidine) (Figure 5) was introduced by Leng et al. in a protocol with EDCI as the coupling reagent [61], and in a subsequent publication, with oxalyl chloride, which was added prior [62]. This protocol, followed by LC-ESI-MS/MS, was applied in human thyroid samples, where 17 FFAs exhibited differences in quantity in thyroid carcinoma samples and para-carcinoma samples [62]. Other reagents that contain a piperazine moiety are Dns-PP (5-dimethylamino-naphthalene-1-sulfonyl piperazine) and Dens-PP (5-diethylamino-naphthalene-1-sulfonyl piperazine) (Figure 5). These structural analogs have been used in twins derivatization protocols for the quantification of FFAs, where Dens-PP is used for labeling the internal standards. Jiang et al. compared this protocol to a non-derivatization protocol and indicated that the detection sensitivities of the analytes were 50 to 1500-fold increased. With their method, they managed to quantify 38 FFAs in rat serum [63]. A similar protocol was used by Xia et al. in 2020 for the quantification of eicosanoids by derivatization with Dns-PP/Dens-PP in plasma samples of type 2 diabetes patients [64]. Additionally, DHPP (*N*,*N*-dimethyl-6,7-dihydro-5H-pyrrolo [3,4-*d*] pyrimidine-2-amine) (Figure 5) is a secondary amine that was used in SCFA and OH-SCFA tagging by Wei et al. [64]. The reported derivatization procedure took place within 3 min under mild conditions and was applied in mouse fecal, serum and liver samples [65].

### 2.3. Aromatic Amines

**Figure 6:**
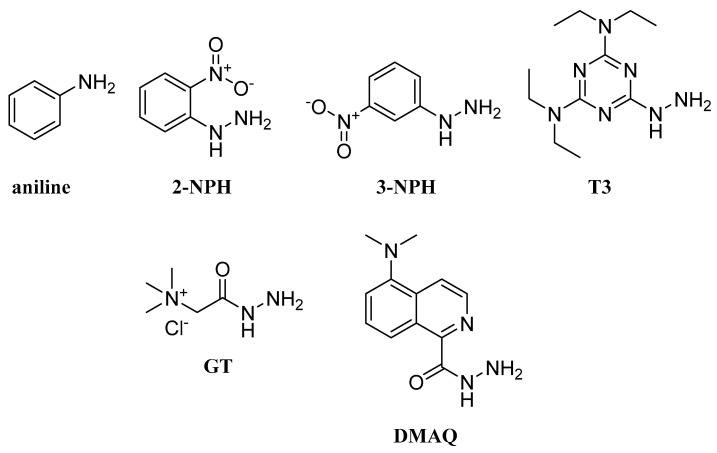
Aromatic amines, hydrazines and hydrazides as derivatization reagents for LC-HRMS analysis of FFAs.

In the literature, aromatic amines have not been extensively employed as labeling reagents for FA LC-MS analysis because of the reduced nucleophilicity of aromatic amines. In 2017, Chan et al. reported an LC/MS/MS method for the determination of 12 SCFAs in human infant stool, using ^12^C- and ^13^C-aniline (Figure 6) [66]. The accurate quantification of the endogenous SCFAs, derivatized by ^12^C-aniline, was based on the calibration of exogenously SCFAs, derivatized by ^13^C-aniline. The authors paid special attention to the residual acetic acid present within the LC/MS system, optimizing the quenching of derivatization agents prior to LC/MS/MS analysis.

### 2.4. Hydrazines and Hydrazides

Hydrazines constitute an additional group of compounds that can readily react with carboxylic acids under mild conditions. For instance, nitrophenylhydrazines (NPH) have appeared as favorable labeling agents for carboxylic acid analytes. Han et al. described a derivatization method that included 3-NPH (Figure 6) for the analysis of SCFAs in human fecal samples, with LODs and LOQs at a femtomole level for 10 SCFAs [67]. A similar protocol was employed in 2020 for the determination of SCFAs and MCFAs in human serum [68]. On the other hand, 2-NPH (Figure 6) has been similarly tested as a labeling agent. At first, Chen et al. developed a protocol for total SCFAs analysis in human serum and their potential variations in blood [69]. Later, this protocol was further improved and used for the quantitation of short, medium, long and very long FAs in human plasma, with good accuracy and sufficient sensitivity in a scale of 0.2–330 fmol for LOD and 2.3–660 fmol for LOQ, for 18 analytes [70]. A structurally different hydrazine derivatization reagent is T3 (2,4-bis(diethylamino)-6-hydrazino-1,3,5-triazine) (Figure 6). Hu et al. reported a UPLC-MS/MS method for the determination of eicosanoids after derivatization with T3. The authors claim that sensitivities of derivatized eicosanoids underwent a 10 to 5000-fold enhancement compared to free eicosanoids, while LOQs for derivatized eicosanoids ranged from 0.05 to 50 pg. The method was applied to biological samples (rat plasma and heart tissue), where more than 40 eicosanoids were identified and quantitated [71]. GT or Girard’s reagent T (trimethylaminoacetohydrazide chloride) (Figure 6) is a hydrazide with a quaternary ammonium salt in its structure, and it can react easily with aldehydes, ketones and carboxylic acids. These characteristics make it an appealing labeling agent for FA analysis. Recently, a new LC-MS/MS method for SCFA quantitation using GT was established, with a sensitivity of GT-derivatized propionate 1.2 × 10^8^ times higher than that of un-derivatized propionate and 100-fold higher than 3-NPH-derivatized propionate. This method was further evaluated using cell culture medium from gut bacteria, namely *E. rectale*, where SCFAs were successfully quantitated [72]. In 2022, Feng et al. reported a LC–MS method for the quantification of SCFAs after derivatization with DMAQ (5-(dimethylamino)-1-carbohydrazide-isoquinoline) (Figure 6). This derivatization, with the use of DMAQ-^13^C/^15^N-tagged SCFAs as internal standards, led to an enhanced detection sensitivity and improved separation of SCFAs. Then, the method was applied in the analysis of the plasma and cecum digesta of neonatal piglets [73].

### 2.5. Bromides

**Figure 7:**
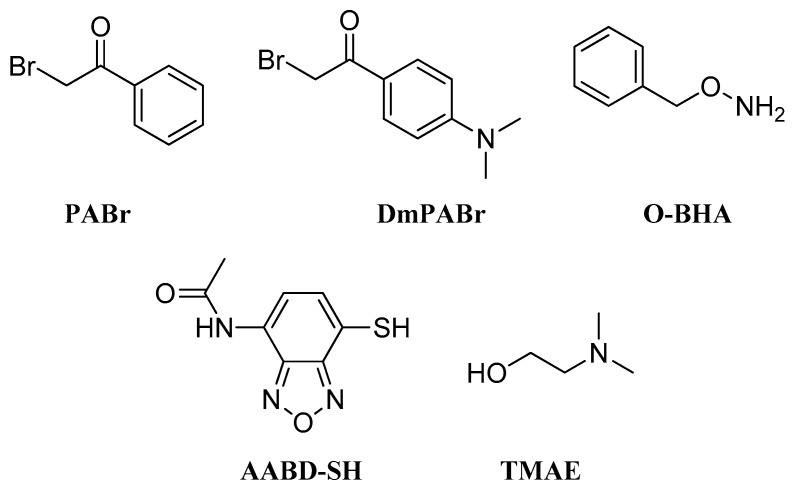
Bromides, hydroxylamines and other derivatization reagents for LC-HRMS analysis of FFAs.

Bromides were used some decades ago as derivatization reagents in HPLC and GC analysis [74,75]. PABr (phenacyl bromide) and its analog DmPABr (dimethylaminophenacyl bromide) (Figure 7) have recently been utilized in LC-MS/MS analysis [76,77]. The PABr protocol was designed for the determination of SCFAs in plasma and feces, showcasing a 200 to 2000-fold improvement in sensitivity compared to the GC method, and was further applied to monitor changes in SCFA quantities in berberine treated hyperlipidemia hamsters [76]. Willacey et al. developed a similar method with DmPABr, adding triethanolamine to the reaction mixture. Their method was applied to HepG2 cells and encompassed different metabolite groups, including amino acids, *N*-acetylated amino acids, acylcarnitines, FAs and tricarboxylic acid cycle metabolites [77].

### 2.6. Hydroxylamines

The importance of SCFAs for human health has made their determination in biological samples necessary and, due to their volatile nature, many labeling agents targeting SCFAs have been established through the years. *O*-ΒHA (*O*-benzylhydroxylamine) (Figure 7), with EDCI as a coupling reagent, was used for this purpose by Zeng et al. in a derivatization procedure in aqueous-organic medium, with LOD for SCFAs at a sub-fmol level [78]. Jaochico et al. also developed a reversed-phase LC-MS/MS method to quantify SCFAs in human plasma and urine samples with *O*-BHA, which showed acceptable accuracy, precision, linearity, recovery and derivatization efficiency [79].

### 2.7. Other Derivatization Reagents

Some other interesting derivatization reagents that have been developed and applied to FA LC-MS analysis are thiol or alcohol compounds. An example of the former is AABD-SH (4-acetoamido-7-mercapto-2,1,3-benzoxadiazole) (Figure 7), a thiol analog that can act as a labeling agent and which has been designed for the profiling of SCFAs. According to the derivatization protocol developed by Song et al., AABD-SH with TPP (triphenylphosphine) and DPDS (2,2′-dipyridyl disulfide) can lead to the formation of stable SCFA derivatives in 5 min at room temperature. This method was used for the measurement of SCFAs quantities in mouse feces, plasma and human breath condensates in order to evaluate its reproducibility [80]. Accordingly, TMAE (2-(dimethylamino)ethanol) (Figure 7) is an alcohol that has been employed for the derivatization of FAs in a stable isotope dilution LC–electrospray ionization/MRM/MS method with LODs in the fmol range. This method was then used in the analysis of FAs in atherosclerotic plaques from carotid arteries [81].

The applications of the carboxyl group derivatization reagents for the determinations of FAs, eicosanoids and FAHFAs are summarized in Table 1.

## 3. Derivatization of Unsaturated Fatty Acids (UFAs)

**Figure 8:**
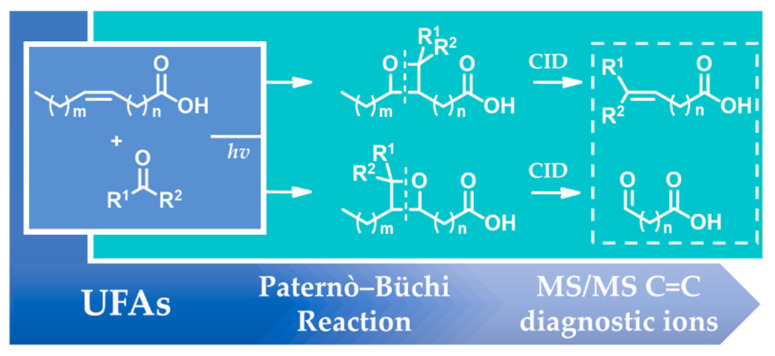
Principle of the combination of the Paternò–Büchi reaction with tandem mass spectrometry (MS/MS) for the determination of the double bond location in UFAs.

**Figure 9:**
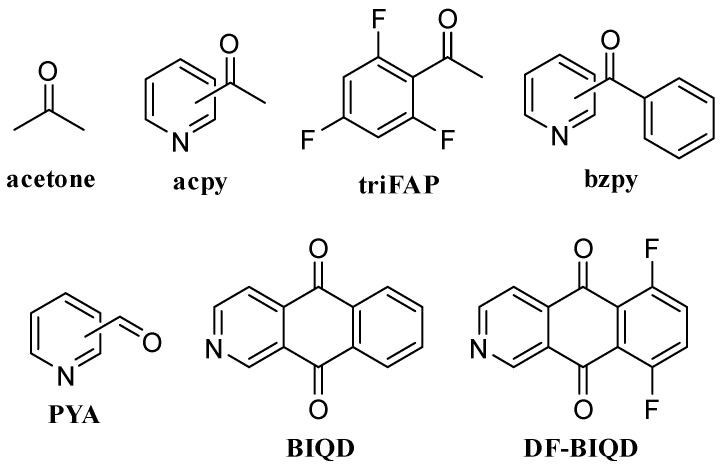
Ketones and aldehydes used for the derivatization of UFAs and determination of their double bond location.

The identification of the position of a double bond in lipids is a long-standing, challenging question. In 2014, Ma and Xia presented a novel method for the fast and sensitive determination of double bond locations in lipids, which combines a Paternò–Büchi (B-P) reaction with tandem mass spectrometry (MS/MS) [82]. The P–B reaction is a classic [2+2] photochemical (UV-facilitated) reaction between an aldehyde or a ketone and an olefin, leading to the formation of an oxetane ring. This first step of the method was followed by a collision-induced dissociation (CID) step in the MS/MS process, leading to the cleavage of the oxetane rings and resulting in the formation of diagnostic ions that were specific to the double bond location (Figure 8). In 2016, Xia’s group reported this online photochemical derivatization analytical methodology for the identification and quantitation of lipid C=C location isomers in complex lipid samples, using acetone (Figure 9) as the derivatization reagent [83]. In 2017, Murphy and co-workers presented the determination of double bond positions in PUFAs, which was based on the UV irradiation of an inline fused silica capillary, when the sample was infused into the ESI source of the instrument [84]. Isopropenyl carboxylate product anions generated during the MS/MS process indicate the location of the double bond, while the product ions resulting from the aldehydic reverse Paternò–Büchi reaction are much less abundant as the length of the chain and the number of double bonds increase [84].

Esch and Heiles studied 2-, 3- and 4-acetylpyridine (acpy) (Figure 9) as the derivatization reagents under irradiation at 254 nm for 30 s and assigned the double bond location in MUFAs and PUFAs [85]. Due to the presence of the pyridine ring, they achieved charge reversal, resulting in 631 times higher ion abundancies in comparison to the abundances of the same compounds prior to the P–B reaction. The functionalization of C=C bonds using 3-acpy followed by MS/MS was also used not only for the study of the C=C bonds location, but also for the identification of hydroxylation sites in lipids [86].

In order to develop next generation P–B reagents with enhanced performance for applications in FA and in general lipid analysis, in 2020, Xia and co-workers studied a variety of acetophenone derivatives [87]. 2′,4′,6′-Trifluoroacetophenone (triFAP) (Figure 9) stood out among the ketones studied, providing an increase in the P–B reaction yield for different types of C=C bonds and high sensitivity (subnanomolar range) for C=C bond identification [87]. More recently, Xia and co-workers investigated the use of various acetyl pyridines, as well as pyridine derivatives of benzophenone, as derivatization reagents enabling charge reversal, and concluded that 2-acetylpyridine (2-acpy) (Figure 9) leads to LODs in the subnanomolar range for MUFAs, PUFAs, as well as conjugated FAs [88]. The same derivatization reagent was applied for the determination of position-specific quantitative deuterium abundancies in bis-allylic deuterated highly unsaturated fatty acids (D-HUFA) (a novel class of drugs stabilized against H abstraction-mediated oxidation) by a shotgun lipidomics analysis [89].

Most recently, to overcome the low reactive rates of P–B derivatization reagents and the low MS response of P–B products, Mao et al. compared a set of P–B derivatization reagents and concluded that 3-pyridinecarboxaldehyde (3-PYA) (Figure 9) leads to high performance [90]. This reagent was successfully applied for either the shotgun or LC-MS analysis of UFAs in a total lipid extract from bovine liver [90]. Most recently, Han et al. developed new P–B derivatization reagents, namely benz[g]isoquinoline-5,10-dione (BIQD) and 6,9-difluorobenzo[g]isoquinoline-5,10-dione (DF-BIQD) (Figure 9), which allow irradiation by visible-light at 405 nm and compact fluorescent lamp visible-light at >400 nm [91].

The ketones and aldehydes used for the derivatization of UFAs, as well as their applications in the analysis of lipids, are summarized in Table 2. In conclusion, the light-mediated P–B derivatization of UFAs, followed by an MS/MS analysis, is a highly useful tool enabling advanced studies in lipid biology and biomarker discovery. Further improvements in this emerging field of lipid analysis and the development of new P–B derivatization reagents may greatly facilitate complex lipid analysis in the coming years.

The advantages offered by the derivatization of FAs, either carboxyl group derivatization or double bond functionalization, are summarized in Table 3. Although the sensitivity of the determination is increased and additional structural information on the lipids under study is usually provided by derivatization, one has to take into consideration that derivatization requires an additional sample pre-treatment step in comparison to the direct determination of underivatized FAs. In the case of eicosanoids and PUFAs, which are usually thermally unstable and easily degradable, the derivatization step must be quick and under low temperature.

## 4. Conclusions

Over the last three decades, advances in MS have made it the most powerful technique for the analysis of FAs, offering high detection sensitivity and selectivity. The derivatization of FAs prior to analysis is often a common and effective approach to increase their ionization efficiency and reduce matrix effect in an LC-MS analysis. A variety of derivatization reagents have been developed over the years, including primary amines, secondary amines, aromatic amines, hydrazines and hydrazides, bromides and hydroxylamines. With the employment of such reagents, the use of a positive mode of ionization is usually achieved due to charge reversal. In addition, the combination of a photochemical P–B reaction with ketones or aldehydes with MS^2^ studies has greatly facilitated the identification of the double bond location in lipids. Various classes of FAs, including saturated FAs, PUFAs and SCFAs, have been successfully analyzed, overcoming volatility issues and helping in the differentiation of FA isomers and the identification of new FAs.

## Figures and Tables

**Table 1:** Summary of FA carboxyl group derivatization reagents and their applications using LC-HRMS.

Derivatization Reagent	Analytical Technique	Instrumental Analysis	Column/Mobile Phase	Sample Preparation— Solvent Extraction/ Cartridge-Column	Sample	Analyte	Ref.
**DMED**	UPLC–ESI–MS/MS (+) ESI mode	ABI/SCIEX 4500 Triple QuadTM coupled to Shimadzu LC-30AD UPLC.	Acquity UPLC BEH phenyl column (2.1 mm × 50 mm, 1.7 μm, Waters) / (Solvent A) HCOOH in H_2_O (0.1%, *v*/*v*) and (Solvent B) ACN/MeOH (7/3, *v*/*v*); flow rate 0.4 mL/min; temperature 40 °C.	Extraction with EtOAc containing 10 μL of BHT (0.10 mM) and 10 μL of 0.5% HCOOH	Serum	Cytochrome P450 metabolites of arachidonic acid	[36]
**DMED**	UHPLC-ESI–MS/MS (+) ESI mode	Shimadzu MS-8040 mass spectrometer (Tokyo, Japan) with an electrospray ionization source (Turbo Ionspray) coupled to a Shimadzu LC-30AD UPLC system (Tokyo, Japan)	Acquity UPLC BEH C18 column (2.1 mm × 50 mm, 1.7 μm, Waters). The mobile phase consisted of (A) HCOOH in ACN/H_2_O (0.1%, 6/4, *v*/*v*) and (B) HCOOH in IPA/ACN (0.1%, 9/1, *v*/*v*); flow rate 0.4 mL/min; temperature 40 °C	Extraction with cold saline solution and ACN containing 0.1% NH_3_/ SAX SPE-cartridge (1 mL, 50 mg) Weltech Co. (Wuhan, China)	Rat tissue Human serum	FAHFAs	[37]
**DMED**	UHPLC/LTQ-Orbitrap MS (+) ESI mode	LTQ-Orbitrap-MS coupled to Shimadzu UHPLC.	Atlantic T3 C18 reverse-phase column (2.1 mm × 150 mm, 3 µm, Waters, Milford, MA, USA) / (Solvent A) 10 mM of aqueous CH_3_COONH_4_ with 0.1% CH_3_COOH, (Solvent B) IPA, (Solvent C) MeOH; flow rate 0.2 mL/min; temperature 40 °C.	Folch method	Colon contents	Short chain FAHFAs	[40]
**DMED**	LC-MS/MS (+) ESI mode	Prominence UFLC (Shimadzu, Kyoto, Japan) coupled to TSQ Quantum Mass Spectrometer System (Thermo Fisher Scientific, San Jose, CA, USA)	Hypersil gold C8 column (50 mm × 2.1 mm, 5 μm; Thermo Fisher Scientific) / Mobile phase A: 20 mM CH_3_COONH_4_, B: MeOH and ACN (1:1); flow rate 0.4 mL/min; temperature 40 °C.	Extraction with ACN	Intestinal contents	SCFAs and hydroxy SCFAs	[41]
**Cholamine**	UHPLC-Q-TOF/MS (+) ESI mode	Agilent 6550 UHD accurate-mass Q-TOF/MS system coupled to Agilent 1290 Infinity LC system (UHPLC, Santa Clara, CA, USA).	Agilent Eclipse XDB-C18 column (2.1 mm × 100 mm, 1.8 μm) / Mobile phase A and B were 0.1% HCOOH-containing H_2_O and 0.1% HCOOH-containing ACN; temperature 40 °C.	Extraction with EtOAc	Serum	Long chain FFAs	[42]
**Cholamine**	LC-MS/MS (+) ESI mode	Xevo TQD triple-quadrupole tandem mass spectrometry coupled to an ACQUITY UPLC system (UPLC-QQQ-MS/MS, Waters Corp., Manchester, UK).	ACQUITY BEH C18 column (150 mm × 2.1 mm i.d., 1.7 μm) / ACN containing 0.1% HCOOH (A, *v*/*v*) and 0.1% aqueous HCOOH solution (B, *v*/*v*); flow rate 0.3 mL/min; temperature 45 °C.	Extraction with cold EtOAc containing 0.5% HCOOH	Serum	n-3 PUFAs and their metabolites	[44]
**DIAAA**	UHPLC-Q-TOF/MS (+) ESI mode	Agilent 6550 UHD accurate-mass Q-TOF/MS coupled to an Agilent 1290 Infinity LC system (UHPLC, Santa Clara, CA, USA)	Waters ACQUITY UPLC HSS T3 column (2.1 mm × 100 mm, 1.8 μm) / Mobile phase A and B were 0.1% HCOOH containing H_2_O and 0.1% HCOOH containing ACN; flow rate 0.3 mL/min; temperature 40 °C.	Extraction with cold MeOH	Serum	SCFAs and LCFAs	[43]
**PFPA**	UPLC-MS/MS (+) ESI mode	5500 QTRAP mass spectrometer (AB Sciex, Foster City, CA, USA) coupled to a Shimadzu LC-30AD UHPLC system (Tokyo, Japan).	Thermo Scientific Accucore pentafluorophenyl (PFP) column (2.1 mm × 15 mm, 2.6 μm) / 0.1% HCOOH in H_2_O/MeOH (3:7, *v*/*v*) as mobile phase A and 0.1% HCOOH in MeOH/IPA (6:4, *v*/*v*) as mobile phase B; flow rate 0.3 mL/min; temperature 40 °C.	Extraction with cold CH_2_Cl_2_/MeOH (2:1, *v*/*v*)	Serum, lung tissue	LCUFAs	[46]
**DAABD-AE**	LC–ESI-MS (+) ESI mode	HP 1090 series II system (Hewlett-Packard GmbH) coupled to an ESI ion trap spectrometer (Esquire 3000+, Brucker Daltonics, Billeria, MA, USA).	Capcellpak C18 (35 mm × 2.0 mm, i.d., 5 µm; Shiseido, Tokyo, Japan) / Mobile phase A: ACN–H_2_O (10:90, *v*/*v*) containing 0.1% HCOOH, and mobile phase B: ACN–H_2_O (90:10, *v*/*v*); flow rate 0.2 mL/min.	Bligh and Dyer method	Rat plasma	FAs	[47]
**AMPP**	LC/ESI-MS/MS (+) ESI mode	Waters Xevo TQ triple quadrupole mass spectrometer coupled to an Acquity UPLC.	Waters Acquity UPLC BEH Shield RP18 (2.1 mm × 100 mm, 1.7 μm) / Solvent A: 100% H_2_O/0.1% HCOOH, solvent B: ACN/0.1% HCOOH; flow rate 0.4 mL/min; temperature 45 °C.	Extraction with MeOH/1N HCl and isooctane	Serum	FAs	[48]
**AMPP**	LC/ESI-MS/MS (+) ESI mode	LTQ Orbitrap mass spectrometer (Thermo Scientific, San Jose, CA, USA) coupled to a Surveyor HPLC system (Thermo Scientific).	C18 reverse phase column (Ascentis Express, 2.7 μm particles, 150 mm × 2.1 mm) / Solvent A: 0.1% glacial CH_3_COOH in H_2_O) and solvent B: 0.1% glacial CH_3_COOH in ACN; flow rate 0.2 mL/min; temperature 23 °C.	Bligh and Dyer procedure/ Strata-X SPE cartridge (30 mg/mL) Phenomenex (Torrance, CA, USA).	Hepatic tissue	Linoleic acid, arachidonic acid, docosahexaenoic acid metabolites	[49]
**AMQ**	UPLC/MS/MS (+) ESI mode	Triple quadrupole 5500 mass spectrometer (AB SCIEX, Redwood City, CA, USA) coupled to a Waters ACQUITY UPLC I-Class system (Waters, Milford, MA, USA).	Waters BEH C18 (2.1 mm × 100 mm I.D., 1.7 μm) UPLC column / H_2_O:HCOOH (1000:1, *v*/*v*; solvent A) and MeOH:HCOOH (1000:1, *v*/*v*; solvent B); flow rate 0.4 mL/min; temperature 50 °C.	Extraction with ethanol	Feces	SCFAs	[51]
**APBQ**	HPLC-ESI-MS/MS (+) ESI mode	API 3000 triple quadrupole-mass spectrometer (Applied Biosystems, Foster City, CA, USA) coupled to an Agilent 1100 HPLC (Agilent Technologies, Santa Clara, CA, USA).	XbridgeTM C18 column (3.5 μm, 150 mm × 2.1 mm id; Waters, Milford, MA, USA) / H_2_O / ACN containing 0.1% *v*/*v* HCOOH; flow rate 0.2 mL/min; temperature 40 °C.	EtOH/SPE cartridge (SOLA-C18, 10 mg/mL, Thermo Scientific, Bellefonte, PA, USA).	Plasma, saliva	FAs	[52]
**2-PA**	UPLC-ESI/MS/MS (+) ESI mode	Waters Xevo TQD triple quadrupole mass spectrometer coupled to a Waters Acquity H Class UPLC system (Waters Co., Milford, MA, USA).	Acquity BEH C18 column (1.7 μm, 2.1 mm × 100 mm) / Mobile phase A and B were 0.1% HCOOH in H_2_O and 0.1% HCOOH in MeOH; flow rate 0.3 mL/min; temperature 40 °C.	Extraction with MeOH	Feces	SCFAs	[56]
**(*R*)-PEA**	LC-DAD-MS (+) API mode	Finnigan LCQ DECA XP MAX (Finnigan, San Jose, CA, USA) quadrupole IT equipped with an API source coupled to a Finnigan Surveyor series liquid chromatograph.	Chromolith RP-18 column (125 4.6 mm) from Merck KGaA / MeOH / H_2_O (90:10); flow rate 0.5 mL/min; temperature 25 °C.	Soxhlet extraction with 1:1 CH_2_Cl_2_/ether solution	Soil samples	FFAs	[57]
**ADMI**	LC–ESI–MS/MS (+) ESI mode	(UPLC-Q/TOF) MS System (H-class UPLC and Synapt G2-Si MS, Waters, Milford, MA, USA)	ACQUITY UPLC BEH C8 column (2.1 mm × 100 mm, 1.8 μm) / Mobile phase A and B were 0.1% HCOOH containing H_2_O and 0.1% HCOOH containing ACN; flow rate 0.4 mL/min; temperature 45 °C.	Addition of H_2_O and extraction with EtOAc	Mouse melanoma samples	2/3 OHUFAS 2/3 OHFAS	[58]
**DMPP**	LC–ESI–MS/MS (+) ESI mode	Triple-quadrupole time-of flight (Q-TOF) mass spectrometer (G6500, Agilent) coupled to an HPLC system (1260 Series LC, Agilent) and an ESI source.	Agilent Zorbax SB-C8, 2.1 mm × 100 mm, 1.8 μm, Santa Clara, CA, USA) / deionized H_2_O (solvent A) and ACN (solvent B); flow rate 0.3 mL/min.	Oasis HLB cartridge (1 cc, Waters Corporation)	Urine	FFAs	[61]
**DMPP**	LC–ESI–MS/MS (+) ESI mode	Triple-quadrupole mass spectrometer (G6410A, Agilent, Santa Clara, CA, USA).	Agilent Zorbax SB-C8, 2.1 mm × 100 mm, 1.8 μm, Santa Clara, CA, USA) / deionized H_2_O (solvent A) and ACN (solvent B); flow rate 0.3 mL/min.	Extraction with CHCl_3_–MeOH (4:1, *v*/*v*)	Human thyroid	FFAs	[62]
**Dns-PP/Dens-PP**	LC-MS/MS (+) ESI mode	MS-8040 triple-quadrupole mass spectrometer (Shimadzu Co., Tokyo, Japan) equipped with an ESI source coupled to a Shimadzu Nexera UPLC system.	Agilent Zorbax Eclipse XDB-C18 column (2.1 mm × 100 mm, 1.8 μm, Agilent Technologies, Santa Clara, CA, USA)/Mobile phase A: 0.1% HCOOH in H_2_O, mobile phase B: MeOH; flow rate 0.4 mL/min; temperature 50 °C.	Extraction with 0.5% HCOOH in H_2_O and EtOAc	Serum	FFAs	[63]
**Dns-PP/Dens-PP**	LC-MS/MS (+) ESI mode	ACQUITY™ ultra performance liquid chromatography (UPLC) coupled to Xevo TQD triple-quadrupole tandem mass spectrometry (UPLC-QqQ-MS/MS, Waters, Manchester, UK).	Hypersil GOLD™ C18 column (150 mm × 2.1 mm i.d., 1.9 μm) / Mobile phase A (0.1% aqueous HCOOH solution) and mobile phase B (ACN/0.1% HCOOH); flow rate 0.4 mL/min; temperature 40 °C.	Oasis HLB cartridge (30 mg, 1 cc, Waters, Manchester, UK)	Plasma	Eicosanoids	[64]
**DHPP**	LC-MS/MS (+) ESI mode	UHPLC coupled to high-resolution orbitrap fusion mass spectrometer (Ultimate 3000 RSLC-Orbitrap Fusion, Thermofisher scientific, Waltham, MA, USA).	Phenomenex polar C18 column (1.6 μm, 2.1 mm × 150 mm) / Mobile phase A: H_2_O/ 0.1% HCOOH and mobile phase B: ACN; flow rate 0.3 mL/min; temperature 30 °C.	Extraction with precooled MeOH	Feces, serum, liver tissue	SCFAs and OH-SCFAs	[65]
**Aniline**	LC-MS/MS (+) ESI mode	AB Sciex QTRAP 5500 hybrid linear ion-trap quadrupole mass spectrometer equipped with a TurboIonSpray source (Applied Biosystems, Foster City, CA) coupled to an Agilent 1290 Infinity LC system (Agilent Technologies, Santa Clara, CA, USA).	Acquity UPLC HSS T3 (1.8 μm, 2.1 mm × 100 mm) / H_2_O and HPLC-grade IPA, both acidified with 0.1% HCOOH; flow rate 0.35 mL/min; temperature 50 °C.	Extraction with 1:1 *v*/*v* ACN/H_2_O	Human stool	SCFAs	[66]
**3-NPH**	UPLC-MS/MS (-) ESI mode	4000 QTRAP triple-quadrupole mass spectrometer (AB Sciex, Concord, ON, Canada) with an ESI source coupled to Ultimate 3000 RSLC system (Dionex Inc., Amsterdam, The Netherlands).	Waters BEH C18 (2.1 mm × 100 mm, 1.7 μm) / H_2_O:HCOOH (100:0.01, *v*/*v*; solvent A) and ACN:HCOOH (100:0.01, *v*/*v*; solvent B); flow rate 0.35 mL/min; temperature 40 °C.	Extraction with 50% aqueous ACN	Feces	SCFAs	[67]
**3-NPH**	LC-MS/MS (-) ESI mode	AB Sciex 3200 QTRAP (Sciex, Milan, Italy) coupled to an HPLC Dionex 3000 UltiMate system (Thermo Fisher Scientific, MA, USA).	Restek Raptor C18 (2.7 μm, 2.1 mm × 100 mm, Bellefonte, PA, USA) / Mobile phase A: H_2_O +0.1% HCOOH and mobile phase B: ACN; flow rate 0.4 mL/min; temperature 35 °C.	Extraction with MeOH/0.05% HCOOH, diluted with deionized H_2_O	Serum	SCFAs and MCFAs	[68]
**2-NPH**	LC-MS/MS (-) ESI mode	Thermo Finnigan Surveyor HPLC-TSQ Quantum Quadrupole mass spectrometer system (Thermo Fisher Scientific Inc., Waltham, MA, USA).	Ascentis^®^ Express Phenyl-Hexyl column (5 cm × 2.1 mm I.D., 2.7 μm, Supelco, Inc., Bellefonte, PA) / 5 mM aqueous CH_3_COONH_4_ (A), IPA (B), and MeOH(C); flow rate 0.2 mL/min; temperature 40 °C.	Saponification with 0.3 M KOH-EtOH	Plasma	SCFAs, MCFAs, LCFAs, VLCFAs	[70]
**T3**	UPLC-MS/MS (+) ESI mode	Agilent 1290 series UPLC system coupled to an Agilent 6490 triple-quadrupole mass spectrometer (Agilent Technologies, Inc. Santa Clara, CA, USA) with an AJS electrospray ionization (AJS-ESI) device.	UPLC BEH C18 column (1.7 μm, 100 mm × 2.1 mm i.d.) / Solvent A: H_2_O (0.2% HCOOH) and solvent B: ACN (0.2% HCOOH); flow rate 0.5 mL/min; temperature 45 °C.	Extraction with MeOH (containing 0.001 M of BHT)/ HLB (30 mg, 1 cc) SPE cartridge	Plasma, heart tissue	Eicosanoids	[71]
**GT**	LC-MS/MS (+) ESI mode	Agilent 6420 triple quadrupole LC/MS (Santa Clara, CA, USA) coupled to an Agilent 1260 Infinity Binary LC (Santa Clara, CA, USA).	Agilent Zorbax HILIC plus column (4.6 mm × 100 mm, 3.5 μm) / Solvent A: H_2_O containing 20 mM of CH_3_COONH_4_ and 20 mM of CH_3_COOH, solvent B: 100% ACN; flow rate 0.5 mL/min.	Dilution with distilled H_2_O	Gut bacteria *E. rectale* culture medium	SCFAs	[72]
**PABr**	LC-MS/MS (+) ESI mode	LC-MS/MS 8050, Shimadzu Corporation, (Kyoto, Japan).	xBridge C18 column (100 mm × 2.1 mm × 3.5 μm, Waters, Milford, MA, USA) / HCOOH:H_2_O (0.1:100, *v*/*v*) (phase A) and MeOH (phase B); flow rate 0.4 mL/min; temperature 30 °C.	Dilution in H_2_O and saturated sodium carbonate solution	Plasma, feces	SCFAs	[76]
**DmPABr**	UPLC-MS/MS (+) ESI mode	AB Sciex QTrap 6500 mass spectrometer (Framingham, MA, USA) coupled to a Waters Acquity UPLC Class II (Milford, MA, USA)	AccQ-tag C18 column (2.1 mm × 100 mm, 1.4 μm, Milford, MA, USA) / Mobile phase A: H_2_O containing 10 mM of NH_4_COOH and 0.1% HCOOH, mobile phase B: 100% ACN; flow rate 0.7 mL/min; temperature 60 °C.	Extraction with H_2_O/MeOH (1:4 *v*/*v*)	HepG2 cells	FFAs	[77]
***O*** **-BHA**	LC-MS/MS (+) ESI mode	5500 triple-quad mass spectrometer (Sciex, Concord, ON, Canada) equipped with Turbospray ESI source coupled to a Shimadzu Nexera X2 UHPLC system (Shimadzu, Kyoto, Japan).	Kinetex C18 (100 mm × 2.1 mm 2.6 μm, Phenomenex, Torrance, CA, USA) / 0.1% HCOOH in H_2_O with 10 mM of NH_4_COOH (solvent A) and 0.1% HCOOH in MeOH: IPA (9:1 *v*/*v*) (solvent B); flow rate 0.4 mL/min; temperature 45 °C.	Extraction with MeOH	Plasma	SCFAs	[78]
**AABD-SH**	LC-MS/MS (+) ESI mode	QTRAP 5500 (ABSciex, Framingham, MA, USA) coupled to a 1290 HPLC instrument (Agilent Technologies, Glostrup, Denmark).	Pursuit 5 C18 (150 × 2.0 mm; Agilent Technologies, Santa Clara, CA, USA) / Mobile phase A (0.1 % HCOOH in H_2_O) and mobile phase B (0.1% HCOOH in ACN); flow rate 0.5 µL/min; temperature 40 °C.	Dilution with H_2_O	Feces, plasma	SCFAs	[80]
**TMAE**	LC-MS/MS (+) ESI mode	Finnigan TSQ Quantum Ultra AM mass spectrometer (Thermo Electron Corporation, San Jose, CA, USA) coupled to a Hitachi L-2130 pump equipped with Hitachi Autosampler L-2200 (Hitachi, San Jose, CA, USA)	Varian Pursuit Diphenyl 3μm column (150 mm × 2 mm i.d., 3μm) / Solvent A: 5 mM of CH_3_COONH_4_ in H_2_O, solvent B: 5mM of CH_3_COONH_4_ in ACN; flow rate 0.5 mL/min; temperature 25 °C.	Hydrolysis with 40% aqueous KOH and extraction with diethyl ether/hexane	Atherosclerotic plaques from carotid arteries	FAs	[81]

**Table 2:** Summary of UFA derivatization reagents and their applications.

Derivatization Reagent	Analytical Technique	Instrumental Analysis	Column/Mobile Phase	Sample Preparation— Solvent Extraction/ Cartridge-Column	Sample	Analyte	Ref.
**Acetone**	MS/MS (-) ESI mode	4000 QTRAP triple quadrupole/linear ion trap (LIT) hybrid mass spectrometer (Sciex, Toronto, Canada)	-	Extraction with MeOH/iso-octane	Rat brain tissue	UFAs	[83]
**Acetone**	MS/MS (-) ESI mode	4000 QTRAP (Applied Biosystems, Thornhill, Ontario, Canada)	-	Bligh−Dyer method	RAW 264.7 cells	PUFAs	[84]
**Acpy**	MS/MS (+) ESI mode	Orbitrap Q Exactive HF instrument (Thermo Fisher Scientific GmbH, Germany)	-	Extraction with MTBE	Mouse brain tissue	MUFAs and PUFAs	[85]
**TriFAP**	MS/MS (+) ESI mode	QTRAP 4500 mass spectrometer (SCIEX, Toronto, Canada)	HILIC column Sigma-Aldrich, MO, USA (150 mm × 2.1 mm, 2.7 μm) / 10 mM of aqueous CH_3_COONH_4_ (A), ACN (B); flow rate 0.2 mL/min; temperature 30 °C.	Extract from Avanti Polar Lipids, Inc. (Alabaster, AL, USA)	Lipid extract from bovine liver	UFAs	[87]
**2-Acpy**	MS/MS (+) ESI mode	X500R QTOF mass spectrometer (Sciex, Toronto, Canada) coupled to a Shimadzu LC-20AD system (Kyoto, Japan)	C18 column Sigma-Aldrich, MO, USA (150 mm × 3.0 mm, 2.7 μm) / H_2_O:ACN (40:60, *v*/*v* with 20 mM ofHCOONH_4_) (A), IPA:ACN (40:60, *v*/*v* with 0.2% FA).(B); flow rate 0.45 mL/min.	Folch method	Human plasma	FAs and UFAs	[88]
**3-PYA**	UHPLC–MS (+) ESI mode	QTRAP 6500 + (AB SCIEX, Framingham, MA, USA) coupled to a Shimadzu LC-30AD system (Shimadzu, Kyoto, Japan)	ACQUITY UPLC BEH C 18 column (Waters, 1.7 μm, 2.1 mm × 100 mm) / H_2_O (A), ACN (B). both mobile phases contained 5 mM of CH_3_COONH_4_; flow rate 0.3 mL/min.	Extract from Aladdin Trading Co., Ltd. (Shanghai, China)	Lipid extract from bovine liver	UFAs	[90]
**BIQD** **DF-BIQD**	LC–MS/MS and MALDI–MS/MS (+) ESI mode	ESI-Q-TOF mass spectrometer (Bruker Daltonics, Billerica, MA, USA) and MALDI-TOF/TOF mass spectrometer (rapiflex TM, Bruker Daltonics, Billerica, MA, USA)	ACQUITY UPLC HSS T3 column (Waters, 3 mm ×100 mm, 1.8 μm) / H_2_O, 0.1% FA (A), ACN/IPA, 9:1, *v*/*v*, 0.1% FA, (B); flow rate 0.25 mL/min; temperature 35 °C.	Extraction with saline solution and ACN	Rat heart, brain, lung, spleen, thymus, kidney, liver and plasma samples	Unsaturated lipids	[91]

**Table 3:** Advantages of derivatization for the analysis of FAs.

Type of derivatization	Sensitivity	Fragmentation
Carboxylic group derivatization	Increase	Increase structural information
Double bond derivatization	Increase	Identification of the double bond location

## Data Availability

Not applicable.
